# Life-Threatening Infantile Diarrhea from Fluoroquinolone-Resistant *Salmonella enteric* Typhimurium with Mutations in Both *gyrA* and *parC*

**DOI:** 10.3201/eid0902.020185

**Published:** 2003-02

**Authors:** Hideo Nakaya, Akihiro Yasuhara, Ken Yoshimura, Yukio Oshihoi, Hidemasa Izumiya, Haruo Watanabe

**Affiliations:** * Kansai Medical University Kohri Hospital, Osaka, Japan; † National Institute of Infectious Diseases, Tokyo, Japan

**Keywords:** *Salmonella* Typhimurium, mutation, fluoroquinolone, dispatch

## Abstract

*Salmonella* Typhimurium DT12, isolated from a 35-day-old infant with diarrhea, was highly resistant to ampicillin, tetracycline, chloramphenicol, streptomycin, gentamycin, sulfamethoxazole/trimethoprim, nalidixic acid, and fluoroquinolones. The patient responded to antibiotic therapy with fosfomycin. Multidrug-resistance may become prevalent in *Salmonella* infections in Japan, as shown in this first case of a patient infected with fluoroquinolone-resistant *Salmonella*.

*Salmonella enterica* serovar Typhimurium (*S*. Typhimurium) is one of the most important causative agents of acute human *Salmonella* gastroenteritis. In particular, *S*. Typhimurium definitive phage type104 (DT104), which has developed multidrug resistance to ampicillin, tetracycline, chloramphenicol, streptomycin, sulfa drugs, and other antibiotics, has quickly become widespread in developed countries and drawn much attention worldwide (*1*–*6*). In European countries, other types of *Salmonella* resistant to fluoroquinolones, including DT104, have been detected, and adequate treatment of infected patients is now a serious issue. We isolated *S*. Typhimurium DT12, highly resistant to fluoroquinolones, from diarrhetic stools from an infant and reported the first clinical infection in Japan (*7*). We describe our analysis of this isolate’s antibiotic susceptibility and drug resistance genes: three point mutations in the region determining quinolone resistance were identified in *gyrA* and *parC*.

## Case Report

The patient was a 35-day-old infant boy with fever, diarrhea, and vomiting. He was born at 38 weeks, weighing 3,296 g. Hyperbilirubinemia developed at 1 week of age. He was fed by both breast milk and formula. The family history was unremarkable. The baby vomited on the night of September 4, 2000, and bloody diarrhea and fever >37°C developed at 3:00 a.m. the next day. His parents consulted the maternity office where he was born, and the obstetrician prescribed oral fosfomycin. When his fever did not subside, the obstetrician referred him to the outpatient department of pediatrics, Kansai Medical University Kohri Hospital, Osaka, Japan. Acute enteritis was diagnosed, and the patient was admitted to the hospital on September 5.

On admission, the infant was 55.0 cm long and weighed 4,536 g. His temperature was 38.6°C, heart rate 162 beats/min, respiratory rate 52/min, and blood pressure 102/palpable mmHg. He was pale and lethargic with cold extremities and cyanosis around the nose and mouth. His anterior fontanelle was 1 cm in diameter without swelling. Small eruptions were observed on his face and neck.

Laboratory evaluation was remarkable for the following: total protein 4.9 g/dL (normal range: 5.0–6.5 g/dL), albumen 2.8 g/dL (normal range: 3.3–4.2 g/dL), C-reactive protein 2.5 mg/dL (normal: <0.3 mg/dL), leukocyte 3,730/mm^3^ (normal range 5,000–19,500/mm^3^), neutrophils 59.4%, lymphocytes 33.0%, sodium ion 135 mEq/L (normal range: 135–147 mEq/L), potassium ion 4.5 mEq/L (normal range: 3.6–5.0 mEq/L), chlorine ion 108 mEq/L (normal range: 98–108 mEq/L), calcium 5.0 mEq/L (normal range 4.2–5.7 mEq/L), blood urea nitrogen 9.8 mg/dL (normal range: 4–15.4 mg/dL), creatinine 0.27 mg/dL (normal range: 0.23–0.6 mg/dL), uric acid 3.6 mg/dL (normal range: 1.4–3.5 mg/dL), and blood sugar 93 mg/dL (normal range: 60–100 mg/dL). *S*. Typhimurium DT12, named KKH712, was isolated from his stool. Liver panel and chemistries were otherwise normal.

Upon admission, fosfomycin by injection was administered for bacterial enteritis. Frequent diarrhea and vomiting decreased. The baby’s body temperature and C-reactive protein level normalized, and his general condition improved. He was discharged on day 14. Outpatient follow-up showed that, with fosfomycin, his stool culture eventually tested negative for *Salmonella*. Stool cultures from his family members (father, mother, sister, and brother) did not show the causative *Salmonella* isolate, and no member of family had diarrhea or took antimicrobial drugs. The family did not have a pet and had not traveled overseas recently. We did not find a route of infection.

## Conclusions

We examined the *S*. Typhimurium DT12 (KKH712) isolated from the patient for drug susceptibility. We determined MICs of 18 antibiotics (ampicillin, cefaclor, cefazolin, ceftazidime, ceftriaxone, imipenem, streptomycin, kanamycin, gentamycin, amikacin, tetracycline, chloramphenicol, sulfamethoxazole/trimethoprim, fosfomycin, nalidixic acid, levofloxacin, ciprofloxacin, and norfloxacin) against the strain by the agar plate dilution method provided by the National Committee for Clinical Laboratory Standards (*8*). The strain, *S*. Typhimurium ATCC13311, was used as a sensitive strain for the comparison, and *Escherichia coli* ATCC25922 was used as the quality control reference strain. Susceptibilities of *S*. Typhimurium DT12 (KKH712) to the different antibiotics are shown in Table 1. MICs were high for ampicillin (MIC:512 µg/mL), streptomycin (512 µg/mL), gentamycin (32 µg/mL), tetracycline (128 µg/mL), chloramphenicol (>128 µg/mL), sulfamethoxazole/trimethoprim (>128 µg/mL), and nalidixic acid (>512 µg/mL), indicating resistance to these antibiotics. The strain was highly resistant to all three fluoroquinolones tested: levofloxacin (8 µg/mL), ciprofloxacin (8 µg/mL), and norfloxacin (16 µg/mL).

**Table 1:** MICs (µg/mL) of 18 antibiotics for *Salmonella* Typhimurium strains (ATCC13311 and KKH712)

Antibiotics	ATCC13311	KKH712
Ampicillin	<0.5	512
Cefaclor	0.5	1
Cefazolin	1	2
Ceftazidime	0.13	0.25
Ceftriaxone	<0.03	0.06
Imipenem	0.13	0.06
Streptomycin	8	512
Kanamycin	1	8
Gentamicin	0.25	32
Amikacin	0.5	1
Tetracycline	1	128
Chloramphenicol	4	>128
Sulfamethoxazole/trimethoprim	1	>128
Fosfomycin	0.5	0.5
Nalidixic acid	4	>512
Levofloxacin	<0.03	8
Norfloxacin	0.06	16
Ciprofloxacin	<0.03	8

Sequence analysis of the *gyrA* and *parC* genes was performed by the method described by Giraud et al. (*9*). In brief, DNA fragments of each gene were amplified in 50-µL reaction mixture by using boiled bacterial suspension with 200 µM of deoxynucleotide triphosphate, 1 µM of the primer pairs, Taq buffer (QIAGEN GmbH, Hilden, Germany), and 2.5 U of Taq DNA polymerase (QIAGEN GmbH). Polymerase chain reaction was run at 93°C for 30 sec, 55°C for 30 sec, and 72°C for 1 min for 35 cycles. The DNA fragments were purified by using MicroSpin Column S-300HR (Amersham Pharmacia Biotech, Piscataway, NJ). Sequence was determined by the method described by Sanger et al. (*10*) in an automatic DNA sequencer (Applied Biosystem 310, Perkin-Elmer Inc., Foster City, CA) by using primer STGYRA1 or STPARC1 for *gyrA* or *parC* fragments, respectively (Table 2). Nucleotide sequences of its gyrA and parC genes were determined and point mutations were detected: Ser83Phe (TCC→TTC) and Asp87Asn (GAC→AAC) in the quinolone resistance–determining region (QRDR) of *gyrA* and Ser80Arg (AGC→CGC) in the QRDR of *parC*.

**Table 2:** Primers for sequence analysis of *gyr*A and *par*C

STGYRA1	5´-TGTCCGAGATGGCCTGAAGC-3´
STGYRA12	5´-CGTTGATGACTTCCGTCAG-3´
STPARC1	5´-ATGAGCGATATGGCAGAGCG-3´
STPARC2	5´-TGACCGAGTTCGCTTAACAG-3´

The World Health Organization has determined that *Salmonella* is reemerging as one of the most important infectious diseases in the world. Drug-resistant *Salmonella* strains, for which infections are increasing worldwide, are of special concern (*1*–*6*). In Japan, quinolone-resistant *S*. Typhimurium strains from domestic cases have been emerging since 1995 (*11*). The phage type of the multidrug-resistant *S*. Typhimurium that we isolated was DT12, not DT104, the prevalent type in developed countries (*12*), which suggests that the strain in this study may differ from the prevalent ones. *Bln*I-digested pulsed-field gel electrophoresis patterns were different between *S*. Typhimurium DT12 KKH712 and typical Japanese isolates of *S*. Typhimurium DT104 (12, data not shown), which also supports the idea that this strain is different. Some multiple drug-resistant *S*. Typhimurium DT12 strains have been reported in Japan, but the frequency of this strain is not as high as that of DT104 (*12*). Recently, other *S*. Typhimurium DT12 strains with high fluoroquinolone resistance in humans were isolated in Japan (pers. comm., H. Izumiya). However, scant data are available on fluoroquinolone resistance of *S*. Typhimurium DT12 originating from cattle in Japan.

The main mechanism of fluoroquinolone resistance by *Enterobacteriaceae*, including *Escherichia coli*, is reported to be several point mutations in the QRDR in the structural gene of DNA gyrase or DNA topoisomerase IV. Analysis of the quinolone resistant gene in the strain obtained from our patient showed three point mutations in QRDR: Ser83Phe (TCC→TTC) and Asp87Asn (GAC→AAC) in QRDR of *gyrA* and Ser80Arg (AGC→CGC) in QRDR of *parC*. These same three mutations have been reported previously in fluoroquinolone-resistant bacteria (*9*,*13*–*15*). However, to the best of our knowledge, this report is the first of a *Salmonella* isolate highly resistant to fluoroquinolones from a clinical case with three point mutations in the QRDR (*16*).

Ampicillin, chloramphenicol, sulfa drugs, and fluoroquinolone have been established as standard first-line therapy for *Salmonella* infections. If the *Salmonella* is a multidrug-resistant strain as in this case, however, all of these antibiotics will be ineffective, and treatment will be difficult. In fact, a previous report describes a patient death after a nosocomial *Salmonella* outbreak in a U.S. hospital (*17*).

Fosfomycin, administered to our patient, has been used to treat various infectious diseases in Japan. This drug is one of the most commonly used antibiotics in Japan because it produces relatively few side effects. In our case, fosfomycin was quite effective against the multidrug- and fluoroquinolone-resistant *Salmonella*; our patient recovered after taking this antibiotic, which is considered relatively safe. Fosfomycin is often administered to babies and children and expected to be effective; however, fosfomycin-resistant *Salmonella* has been reported in Japan (*18*). In Japan, fosfomycin was approved for use in animals in 1986. Thus far, fosfomycin-resistant *Salmonella* has not increased. We must be very careful not to overuse this antibiotic and thereby introduce *Salmonella* strains resistant to fosfomycin as has happened with the fluoroquinolones.

The emergence of fluoroquinolone-resistant *Salmonella* in European countries is attributed to the use of fluoroquinolones in livestock and the accompanying natural selection of the resistant strain. In Japan, fluoroquinolones were approved for use in animals in 1991. Fluoroquinolones tend to be used more frequently in Japan than in Europe. Persons in Japan, then, are at risk of having more infectious diseases caused by fluoroquinolone-resistant *Salmonella* and other bacteria. Surveillance for antimicrobial resistance of *Salmonella* should be continued, particularly to monitor the emergence of strains with high fluoroquinolone resistance from humans and livestock.
